# Impact of MMC-assisted glycemic control assistant on biochemical indicators of glucose metabolism in type 2 diabetes: A retrospective study

**DOI:** 10.5937/jomb0-60285

**Published:** 2026-01-06

**Authors:** Peipei Shen, Hongjiao Yang

**Affiliations:** 1 The People's Hospital of Pizhou, Department of Endocrine and Metabolic Diseases, Pizhou, Xuzhou, Jiangsu, China

**Keywords:** fasting plasma glucose, HbA1c, postprandial glucose, glycemic control, type 2 diabetes, biochemical monitoring, glukoza u plazmi na gladno, HbA1c, postprandijalna glukoza, glikemijska kontrola, dijabetes tipa 2, biohemijsko praćenje

## Abstract

**Background:**

Effective glycemic management in type 2 diabetes remains challenging due to limited patient self-management and fragmented care. The National Standardized Metabolic Management Center (MMC)-assisted glycemic control assistant is a novel digital platform that integrates real-time monitoring, education, and treatment adjustment. This study evaluated its impact on biochemical markers and patient behaviors compared with routine care.

**Methods:**

A retrospective study was conducted on 160 patients with type 2 diabetes, including 95 who received MMC-assisted digital management and 65 who received routine care. Key biochemical parametersfasting plasma glucose (FPG), 2-hour postprandial glucose (2hPG), and glycated hemoglobin (HbA1c)were measured using standardized enzymatic assays. Dietary behavior and diabetes self-management were also assessed using validated scales.

**Results:**

At baseline, groups were comparable in clinical and behavioral characteristics. After intervention, the MMC group achieved greater improvements: FPG decreased by 10.1% (7.42± 2.43 vs. 8.30± 2.66 mmol/L, P= 0.032), 2hPG by 17.5% (10.23± 3.21 vs. 12.39± 3.50 mmol/L, P &lt; 0 .0 0 1 ), and HbA1c by 9.0% (7.26 ± 2.05 % vs. 7.98± 2.34% , P = 0.041). Significant gains were also observed in dietary behavior and adherence to glucose monitoring, medication, and exercise.

**Conclusions:**

The MMC-assisted glycemic control assistant enhances glycemic control and promotes healthier behaviors in type 2 diabetes. These findings support its clinical utility and highlight the potential for broader integration of digital tools into standardized chronic disease management.

## Abstract (sr)

**Uvod:**

Efikasno upravljanje glikemijom kod dijabetesa tipa 2 ostaje izazovno zbog ograničenog samostalnog upravljanja pacijentima i fragmentirane nege. Asistent za kontrolu glikemije potpomognut Nacionalnim standardizovanim centrom za upravljanje metabolizmom (MMC) je nova digitalna platforma koja integriše praćenje u realnom vremenu, edukaciju i prilagođavanje lečenja. Ova studija je procenila njen uticaj na biohemijske markere i ponašanje pacijenata u poređenju sa rutinskom negom.

**Metode:**

Retrospektivna studija je sprovedena na 160 pacijenata sa dijabetesom tipa 2, uključujući 95 koji su primali digitalno lečenje uz pomoć MMC-a i 65 koji su primali rutinsku negu. Ključni biohemijski parametri - glukoza u plazmi na gladno (FPG), glukoza nakon 2 sata (2hPG) i glikovani hemoglobin (HbA1c) - mereni su korišćenjem standardizovanih enzimskih testova. Dijetetsko ponašanje i samokontrola dijabetesa takođe su procenjeni korišćenjem validiranih skala.

**Rezultati:**

Na početku, grupe su bile uporedive po kliničkim i bihejvioralnim karakteristikama. Nakon intervencije, MMC grupa je postigla veća poboljšanja: FPG se smanjio za 10,1% (7,42 ± 2,43 naspram 8,30 ± 2,66 mmol/L, P = 0,032), 2hPG za 17,5% (10,23 ± 3,21 naspram 12,39 ± 3,50 mmol/L, P &lt; 0,001) i HbA1c za 9,0% (7,26 ± 2,05 % naspram 7,98 ± 2,34 % , P = 0,041). Značajni dobici su takođe primećeni u ishrani i pridržavanju praćenja glukoze, lekova i vežbanja.

**Zaključak:**

Asistent za kontrolu glikemije potpomognut MMC-om poboljšava kontrolu glikemije i promoviše zdravije ponašanje kod dijabetesa tipa 2. Ovi nalazi podržavaju njegovu kliničku korisnost i ističu potencijal za širu integraciju digitalnih alata u standardizovano upravljanje hroničnim bolestima.

## Introduction

Type 2 diabetes has become the most common chronic metabolic disease in clinical practice today, especially with the increasing aging population worldwide, leading to an increasing prevalence of diabetes year by year. The disease is mainly caused by insulin deficiency and abnormal glucose-raising hormones leading to elevated blood glucose levels, accompanied by abnormalities in protein and lipid metabolism. Long-term control of diet, blood glucose, and weight is required to stabilized disease conditions. Poor control of diabetes can lead to neurological disorders, cardiovascular and cerebrovascular diseases, which eventually result in compromising of mortality and mobility [1]. However, most of the diabetic patients are in old age and lack of the awareness of diabetes, some even suffer from poor cognitive ability and memory. A previous survey analysis [2] showed that the control of diabetes is still the main challenge faced by patients with the disease worldwide, and about 50% of patients with type 2 diabetes achieve blood glucose control. Therefore, it is crucial to provide a convenient and effective management model for these patients.

The National Standardized Metabolic Management Center (MMC) is founded in 2016 by the Chinese Medical Doctor Association. This platform provides a comprehensive series of services including registration, tests, evaluation, prescriptions, and health education in one center. The MMC also provides services outside the hospital, such as medication reminder and doctor-patient communication. In short, the MMC program provides effective diagnosis and treatment, as well as comprehensive management for metabolic diseases [3]. Ke et al. [4] reported that MMC-assisted comprehensive health education can significantly reduce blood glucose levels and improved the quality of life in diabetic patients. With the development of electronic technology, some software was introduced to disease management. For example, Han CY et al. [5] found that Huayi blood glucose steward is beneficial to blood glucose control, diabetes self-management and other metabolic indicators. MMC-assisted glycemic control assistant can provide benefits on diabetes management and delay progression. However, unlike such stand-alone applications, the MMC-assisted glycemic control assistant is embedded within the standardized MMC framework and directly links patient self-monitoring data with clinical decision-making in real time. This integration enables individualized treatment adjustment, continuous follow-up, and comprehensive education that go beyond conventional management approaches. Despite its promise, the MMC program is relatively new, and evidence on the clinical effectiveness of the MMC-assisted glycemic control assistant in routine practice remains limited. Therefore, its potential impact on glycemic control and patient self-management warrants further investigation.

In this study, we compared key biochemical markers of glucose metabolism and behavioral outcomes between patients managed with the MMC-assisted glycemic control assistant and those receiving routine care. We hypothesized that MMC-assisted management would lead to greater improvements in glycemic control (as reflected by FPG, 2hPG, and HbA1c) and enhanced dietary and self-management behaviors compared with conventional management. This study therefore aimed to evaluate the clinical effectiveness of MMC-assisted glycemic control assistant in patients with type 2 diabetes.

## Materials and methods

### Study population

A retrospective analysis was conducted on 160 patients with type 2 diabetes admitted to our hospital from August 2021 to April 2023. Patients were categorized into a research group (*n* = 95) and a control group (*n* = 65) based on the management they received. Patients who received the MMC-assisted glycemic control assistant were included in the research group, and patients who received routine management were included in the control group. This study has been approved by the Ethics Committee of our hospital, and all procedures are in accordance with the ethical standards set forth in the 1964 Helsinki Declaration and its later amendments. As a retrospective cohort study, patient identification information was hidden and informed consent was not required.

Inclusion criteria: (1) All met the diagnostic criteria for type 2 diabetes in »Classification and Diagnosis of Diabetes« [6]; (2) Complete medical records; (3) All have smartphones and can use them proficiently; (4) Disease duration more than 3 years; (5) Normal cognitive ability and autonomy.

Exclusion criteria: (1) Presence of immune system diseases, malignant tumors, or blood diseases; (2) Comorbidity with mental disorders or hearing and visual impairments; (3) Merge brain tumors or cerebral infarction; (4) Pregnant women with diabetes or diabetes are in critical condition.

### Patient managements

Control group: Routine management was adopted. (1) General information, disease history, family history and tests results are collected when patients visit the clinic. (2) Diagnosis and prescriptions are made by the physicians. (3) Health education about diabetes is provided for the patients. (4) Regular telephone follow-up. Consultation services were also provided.

Research group: MMC-assisted glycemic control assistant management was adopted. (1) Create a personal account for the patient on their visit for the clinic. Collect and upload the general information, disease history, family history and self-management. (2) Patients undergo various examinations and tests on diabetes, other metabolic condition, and function of major organs such as heart, kidney, and liver. Upload the tests results to the MMC platform. (3) Patients learn to use the glycemic control assistant on their mobile phone for self-management. The assistant provides functions including health consultation, outpatient appointment, disease knowledge, doctor updates, follow-up reports, and reminders for monitoring and medication. (4) The glycemic control assistant automatically generated dynamic curves for physicians to view and adjust treatment plans

### Data collection

### Biochemical parameter assessment

Fasting plasma glucose (FPG), 2-hour postprandial plasma glucose (2hPG), and glycated hemoglobin (HbA1c) levels were measured in both groups before and after the intervention. These parameters were analyzed using a fully automated biochemical analyzer (BS-280, Mindray), employing the glucose oxidase enzymatic method in accordance with standard laboratory protocols.

### Evaluation of dietary behavior

Patients' dietary behaviors were assessed at baseline and at the two-month follow-up using a self-developed dietary behavior questionnaire. This instrument comprises three dimensions: special diet (assessing patients' knowledge of and adherence to specific dietary requirements), daily diet (evaluating meal regularity and nutritional balance), and cooking methods (assessing the use of healthy preparation techniques and fresh ingredients). The questionnaire contains six items, each scored on a five-point Likert scale ranging from »completely inconsistent« to »completely consistent.« Each subscale has a maximum score of 10, with higher scores indicating better dietary behavior. The instrument demonstrated good psychometric properties, with a Cronbach's coefficient of 0.821, test-retest reliability of 0.857, and content validity index of 0.806.

### Assessment of diabetes self-management

Self-management behaviors were measured using the Diabetes Self-Care Scale (DSCS) at both baseline and the two-month follow-up. The DSCS includes six domains: control of blood glucose abnormalities (maximum 20 points), foot care (25 points), blood glucose monitoring (20 points), regular diet (30 points), medication adherence (15 points), and regular exercise (20 points). The scale comprises 26 items rated on a five-point Likert scale from »did not do at all« to »completely did,« with higher scores indicating stronger self-management capabilities. Interpretation of total scores classifies self-management ability as poor (1-5), moderate (6-10), or strong (11). The DSCS has demonstrated robust reliability and validity, with a Cronbach's of 0.835, test-retest reliability of 0.861, and content validity index of 0.827.

### Statistical analysis

The data were analyzed using Statistic Package for Social Science (SPSS) 25.0 statistical software (IBM, Armonk, NY, USA). Count data were expressed as percentages and analyzed using the chi-square test. Measurement data that followed a normal distribution were expressed as mean ± standard deviation (χ±s) and analyzed using the t-test. If the data did not follow a normal distribution, statistical analysis was performed after transforming the variables to a normal distribution. In addition, to minimize the influence of potential confounders such as age, sex, education level, and disease duration, we performed multivariate linear regression analyses when comparing post-intervention outcomes between groups. Variables that showed imbalance at baseline or were clinically relevant were entered as covariates. *P* value less than 0.05 was considered statistically significant.

## Results

### Baseline characteristics

As shown in Table 1, there were no statistical differences in baseline characteristics (sex, age, disease duration, body mass index, education level, smoking, drinking, and family history of diabetes) between the two groups (P>0.05).

**Table 1:** Comparison of clinical data between the two groups.

Clinical data	Research group (n=95)	Control group (n=65)	*χ^2^/t*	* P *
Sex				
Male	53 (55.79%)	38 (58.46%)	0.030	0.863
Female	42 (44.21%)	27 (41.54%)		
Age (years)	57.48±6.23	57.01±6.55	0.458	0.648
Duration (years)	8.59±2.34	8.23±2.07	1.032	0.304
Body mass index (kg/m^2^)	23.40±1.97	23.66±1.85	0.836	0.404
Education level				
Junior high school and below	21 (22.11%)	17 (26.15%)	0.379	0.828
High school	42 (44.21%)	28 (43.08%)		
College and above	32 (33.68%)	20 (30.77%)		
Smoking	57 (60.00%)	37 (56.92%)	0.051	0.822
Drinking	63 (66.32%)	41 (63.08%)	0.064	0.800
Family history	38 (40.00%)	29 (44.62%)	0.175	0.676

### Blood glucose control

FPG and 2hPG are common markers for assessing glucose and insulin tolerance, while HbA1c reflects long-term glycemic control. At baseline, no significant differences were observed between the two groups. After intervention, patients in the MMC-assisted group showed significantly lower FPG (7.42±2.43 vs. 8.30±2.66 mmol/L, P=0.032), 2hPG (10.23±3.21 vs. 12.39±3.50 mmol/L, P<0.001), and HbA1c (7.26±2.05% vs. 7.98±2.34%, P=0.041) compared with the control group, indicating improved glycemic control with MMC-assisted management. Details are presented in Table 2.

**Table 2:** Comparison of blood glucose index between the two groups (χ̄±s).

Group	FPG (mmol)		2hPG (mmol)		HbAlc (%)	
	Before	After	Before	After	Before	After
Research group (n=95)	9.36±3.02	7.42±2.43	15.18±4.36	10.23±3.21	9.36±3.02	7.26±2.05
Control group (n=65)	9.25±3.07	8.30±2.66	15.42±4.15	12.39±3.50	9.25±3.07	7.98±2.34
* t *	0 222	2.165	0.361	3.979	0.222	2.059
* P *	0.825	0.032	0.718	<0.001	0.825	0.041

### Dietary behavior

Dietary behavior was assessed across three dimensions: adherence to special dietary requirements, regularity and balance of daily meals, and use of healthy cooking methods with fresh ingredients. At baseline, no significant differences were observed between the two groups. After intervention, patients in the MMC-assisted group achieved significantly higher scores in special diet (7.96±1.14 vs. 7.02±1.30, P<0.001), daily diet (8.13± 1.21 vs. 7.64±1.45, P=0.022), and diet cooking (8.89±1.01 vs. 8.03±1.23, P<0.001), reflecting more favorable dietary behavior patterns compared with the control group. Details are shown in Table 3.

**Table 3:** Comparison of dietary behavior between the two groups (χ̄±s).

Group	Special diet		Daily diet		Dietary cooking	
	Before	After	Before	After	Before	After
Research group<br>(n=95)	5.34±1.68	7.96±1.14	6.02±1.85	8.13±1.21	6.74±2.05	8.89±1.01
Control group<br>(n=65)	5.41±1.70	7.02±1.30	5.95±1.90	7.64±1.45	6.90±2.13	8.03±1.23
*t*	0.258	4.837	0.233	2.319	0.477	4.838
*P*	0.797	<0.001	0.817	0.022	0.634	<0.001

### Self-management behavior

Self-management was evaluated using the Diabetes Self-Care Scale before and after intervention. At baseline, scores were comparable between groups. After intervention, the MMC-assisted group demonstrated significantly higher scores in blood glucose monitoring (17.20 ± 2.73 vs. 15.36 ± 3.02, P<0.001), regular diet (24.10 ± 1.85 vs. 21.69 ± 2.14, P<0.001), medication adherence (12.03 ± 1.45 vs. 11.44 ± 1.98, P=0.044), and regular exercise (16.52 ± 1.41 vs. 15.71 ± 1.80, P=0.003). No significant difference was observed in blood glucose control or foot care. These findings indicate that MMC-assisted management enhanced multiple aspects of self-care behavior in patients with diabetes. Details are presented in Table 4. 

**Table 4:** Comparison of DSCS scores between the two groups (χ̄±s).

Group	Blood glucose <br>monitoring		Regular diet		Adherence to <br>medication		Blood glucose <br>control		Regular exercise		Foot care	
	Before	After	Before	After	Before	After	Before	After	Before	After	Before	After
Research <br>group <br>(n=95)	13.58±3.52	17.20±2.73	19.38±3.46	24.10±1.85	9.65±2.21	12.03±1.45	15.03±2.17	18.05±0.67	13.14±2.38	16.52±1.41	18.22±3.07	22.09±1.10
Control <br>group <br>(n=65)	14.11±3.76	15.36±3.02	18.75±3.55	21.69±2.14	9.53±2.17	11.44±1.98	14.86±2.05	17.82±0.83	13.65±2.51	15.71±1.80	18.73±3.14	21.74±1.47
* t *	0.893	3.93	1.128	7.389	0.327	2.035	0.515	1.819	1.285	3.028	1.020	1.607
* P *	0.374	<0.001	0.261	<0.001	0.744	0.044	0.607	0.071	0.201	0.003	0.309	0.111

## Discussion

Diabetes, as a chronic disease, often requires long-term blood glucose monitoring and medication/insulin therapy. Self-management is dynamic process in managing chronic diseases. Without establishing good self-management, the patients could ultimately lead to poor blood glucose control and complications [7]. A previous study on the management of type 2 diabetes found that among patients with a disease duration of 1-4 years, 5-9 years, and 10 years or more, the success rates of blood sugar control were reduced to 31.4%, 27.1%, and 15.3% respectively [8]. Therefore, improving glycemic control in diabetes patients becomes crucial.

MMC is a program led by Shanghai Ruijin Hospital and conducted in mainland China, aiming to provide standardized management for metabolic diseases [9]. This study based on the combination of MMC and glycemic control assistant, using an online platform to connect patients and hospitals, and establish a real-time communication and collaboration bridge. Our results revealed that the main advantage of MMC-assisted glycemic control assistant is to establish a more effective model of chronic diseases management including diabetes. The superior glycemic outcomes observed in the MMC-assisted group may be attributed to the platform's integration of real-time data monitoring, personalized treatment adjustment, and continuous patient education. These features likely enhanced adherence to lifestyle recommendations and medication, thereby improving biochemical indicators beyond what routine care could achieve. Such mechanisms are consistent with prior studies showing that digital health interventions promote patient engagement and long-term behavioral change. A study using convenient sampling method also proved that FPG, 2hPG, and HbA1c levels significantly decreased after MMC intervention [10]. Using diabetes mobile applications for diabetes self-management is one of the emerging strategies to control blood glucose levels and maintain the health of diabetes patients. As found by Gosak et al compared to the control group that did not use mobile applications, the intervention group of type 2 diabetes patients experienced a decrease in blood glucose and HbA1c levels during their first visit [11]. This article is based on the joint blood sugar control assistant on the MMC, using a modern Internet platform to connect patients and hospitals, building a real-time communication and joint bridge. It not only allows medical staff to fully understand changes in patients' conditions and blood sugar fluctuations, but also provides a basis for adjusting treatment plans and management content. Patients can also use this method to consult at any time, even after discharge, they can continue to receive comprehensive disease management services, effectively enhance self-management effectiveness, reduce blood sugar fluctuations, and promote stability of blood sugar indicators such as FPG, 2hPG, HbAlc [12] [13] [14].

In recent years, people's dietary habits in China have gradually shifted towards high-fat, high-salt, and low-fiber diets, directly increasing the risk factors for diabetes and even the mortality of diabetes. As mentioned in a clinical study [15] [16], scientific dietary regulation is helpful in maintaining stable blood glucose levels and is equally important in preventing or delaying complications and improving quality of life. However, in previous routine management of diabetes patients, clinical attention to the impact of dietary behavior on the condition has often been overlooked, and little emphasis has been placed on dietary management, resulting in low dietary compliance. At the same time, many patients find that strict adherence to diet is the most challenging aspect of controlling diabetes, and compliance with dietary recommendations is still unsatisfactory [17]. Failure to control dietary behavior may directly affect their food intake, leading to an increasing incidence of obesity- related diseases such as diabetes and metabolic disorders including insulin resistance [18]. Therefore, early correction of dietary behavior in diabetic patients plays an important role.

The ability to self-manage refers to a series of protective behaviors that patients adopt in their daily lives to control their condition and promote health. If diabetes patients have a high level of self-management ability, they can actively control their blood sugar levels and delay the progression of the disease. However, some scholars have suggested [19] that although increasing knowledge and skills through diabetes education and adherence to certain guidelines are important aspects of diabetes self-management, there may be other factors that affect poor blood glucose control and non-adherence to treatment plans in individuals from low-income backgrounds. In a cross-sectional study [20], it was found that 28.1% of patients had never had regular blood glucose testing, and among the results of the self-management scale, the average score for self-monitoring blood glucose was the lowest. This indicates that diabetes patients may lack relevant skills or rarely engage in self-care activities, resulting in poor self-management ability in blood glucose self-monitoring. Due to certain objective factors, such as educational level or poor self-control, diabetes patients are unable to maintain good self-management ability. Alshahri et al. [21] also proposed that poor self-management ability is related to poor blood glucose control. Therefore, timely and effective measures are crucial for intervening to enhance diabetes patients' self-management ability.

As mentioned by a clinical study [22], mobile health applications have become useful tools to supplement diabetes self-management. Kebede et al. [23] also believe that diabetes applications help patients effectively improve blood glucose control by enhancing diabetes knowledge, self-management ability, adherence to medication, and healthy lifestyle. As found in a study, the use of diabetes applications for self-management in diabetes patients is positively correlated with self-care behaviors [24]. Interestingly, no significant improvement was observed in foot care behaviors. This may reflect the relatively short study duration and the fact that foot care is often underrecognized by patients without overt complications. Moreover, the current MMC modules place greater emphasis on glucose monitoring, diet, and medication adherence, while foot care-specific education is less prominent. Future iterations of the program could incorporate targeted foot care modules, including visual education materials and remote monitoring, to address this gap and reduce the risk of diabetic foot complications.

This study effectively improved patients' cognitive abilities by using the MMC-assisted blood sugar control assistant to send disease-related videos and information at regular intervals, enabling patients to fully understand and master the methods of self-monitoring their condition. In addition, online knowledge lectures and counseling services are beneficial for correcting patients' erroneous behaviors in daily life, promoting their self-management ability, and providing assurance for stable blood sugar levels and disease stability [25] [26]. However, this study still has several limitations. As a retrospective cohort study, it may not eliminate potential confounding factors. However, the study collected general patient information as much as possible and found comparability between the two groups. In addition, the selection of assessment scales to evaluate patient diet behavior and self-management ability may be influenced by subjective factors. However, this study also included the use of objective measurement tools, such as blood glucose indicators, to provide objective evidence for the results. In conclusion, although this study has the limitations, it still provides substantial support for the subsequent use of MMC-assisted glycemic control assistants in managing patients. In future clinical studies, it is expected to adopt more rigorous designs, expand the sample size, and extend the study period to obtain more scientific results and promote the development of this management model.

In summary, MMC-assisted glycemic control assistants play an important role in improving blood glucose levels in diabetic patients. They also help control patient diet behavior and enhance their self-management ability, providing assurance for stabilizing the condition as quickly as possible. Beyond clinical effectiveness, the MMC-assisted model also has potential advantages in cost-effectiveness and scalability. By reducing the need for frequent in-person visits, optimizing physician time through automated data integration, and improving patient adherence, the program may lower long-term healthcare costs associated with diabetes complications. Moreover, because the platform is digital, it can be readily scaled to community health centers and primary care settings, providing standardized diabetes management across diverse healthcare environments. This scalability aligns with current priorities for sustainable chronic disease management in resource-limited settings.

## Dodatak

### Conflict of interest statement

All the authors declare that they have no conflict of interest in this work.
